# Comparative Analysis of the Morphological, Physiological, Proteomic, and Metabolic Mechanisms of the “Biloxi” Blueberry Response to Shade Stress

**DOI:** 10.3389/fpls.2022.877789

**Published:** 2022-05-03

**Authors:** Yaqiong Wu, Zhengjin Huang, Chunhong Zhang, Chong Shi, Lianfei Lyu, Weilin Li, Wenlong Wu

**Affiliations:** ^1^Jiangsu Key Laboratory for the Research and Utilization of Plant Resources, The Jiangsu Provincial Platform for Conservation and Utilization of Agricultural Germplasm, Institute of Botany, Jiangsu Province and Chinese Academy of Sciences, Nanjing, China; ^2^Co-Innovation Center for Sustainable Forestry in Southern China, Nanjing Forestry University, Nanjing, China

**Keywords:** growth, physiology, flavonoid, differentially expressed proteins, correlation

## Abstract

Blueberry is an important small berry crop in economic forests. In hot summers, the top tip of blueberry often burns and withers due to water loss. Therefore, this study subjected blueberry to shading treatment in the summer to study the effects of different shading treatments on the growth, morphology, physiology and protein levels of the plant. The results showed that the 50% shading (T1) treatment yielded the highest average increases in plant height, crown width, and ground diameter of blueberry. Under the 80% shading (T2) treatment, the cells of the leaves dissolved, the morphology was incomplete, the vascular bundles disappeared, and no supporting skeleton was detected. As demonstrated by physiological and biochemical data and the proteome expression levels, the T1 shading treatment was beneficial to the growth of blueberry and significantly enriched the photosynthetic pathway and flavonoid biosynthesis. An analysis of the interaction network of differentially expressed proteins indicated that trans-cinnamate 4-monooxygenase (C4H, CYP73A), naringenin 3-dioxygenase (F3H) and bifunctional dihydroflavonol 4-reductase/flavanone 4-reductase (DFR) exhibited high connectivity and mutual regulation. In short, 50% shading can improve the growth index of blueberry and lead to an enrichment of flavonoid biosynthesis. This study provides a scientific basis for the breeding and summer protection of blueberry seedlings.

## Introduction

Blueberry (*Vaccinium corymbosum* L.) is a perennial shrub of the genus *Vaccinium* in Ericaceae and an economically important woody fruit crop (Wu et al., 2022). It was originally produced in North America and then began to be cultivated and produced in more than 30 countries, such as Canada, China and Japan (Gupta et al., 2015). In recent years, the cultivated area of blueberry in China has exhibited continuous expansion from Northeast China to East China, South China, Southwest China and other places (Li et al., 2021). By the end of 2020, the cultivated area of blueberry in China reached 66,400 hm^2^ and yielded a total output of 347,200 tons and a fresh fruit output of 234,700 tons, which makes China the major exporter of blueberry in Asia (Yu et al., 2020; Li et al., 2021). Blueberry fruits are small, dark blue or deep purple berries with a delicate pulp, moderately sweet and sour tastes, and high nutritional value. Its fruits contain flavonoids, anthocyanins and other antioxidant substances (Li et al., 2016; Lin et al., 2020). Therefore, blueberry is considered the most representative healthy berry and has received wide attention worldwide (Siddiq and Dolan, 2017; Wang et al., 2017). In addition, blueberry leaves are rich in polyphenols, flavonoids, anthocyanins, and other active ingredients and thus exert certain antioxidant, anti-inflammatory, and bactericidal effects, including cardiovascular protection and other health benefits (Zifkin et al., 2012; Li et al., 2013; Routray and Orsat, 2014; Ge et al., 2019). Blueberry is one of the world’s four most important small berry fruit trees with high economic value, and its planting area is increasing yearly. As a byproduct of the blueberry planting process, blueberry leaves also have high development and utilization value (Günther et al., 2020). Research has shown that nearly 60% of blueberry production occurs during periods of yield instability, which is jointly influenced by genetic and environmental factors (Petridis et al., 2018). One possible reason for this finding is that limited photosynthetic efficiency hinders the assimilation, storage and distribution of carbon, resulting in blueberry tissue being unable to accumulate starch and other energy storage substances. The photosynthetic efficiency level directly determines the productivity of blueberry (Huang et al., 2018; Petridis et al., 2018).

Plant growth and developmental processes significantly related to the light environment, such as morphogenesis, physiological metabolism, material accumulation and gene regulation, are affected by external light intensity (Sims and Pearcy, 1991; Zoratti et al., 2014; Smith and Malladi, 2017; Wang et al., 2019; Dennis et al., 2020). Because photosynthesis is the main driving force of plant nutrient accumulation, plants produce organic matter through photosynthesis, and this matter is transported to different metabolic sinks and transformed into different metabolites (Li et al., 2017). In the plant response to light intensity, the shading of plants appropriately can effectively prevent excessive energy formation in seedlings, effectively promote the growth and development of plants and maintain their morphology (Wu et al., 2016, 2020). However, long-term shading will also affect the growth and development of plants; thus, moderate shading is more conducive to plant regeneration, reproduction and progressive succession (Feng and Van Kleunen, 2014). In summer, excessive light changes the morphological structure, growth and development, and physiological and biochemical characteristics of plants to maintain their normal life activities (Hussain et al., 2018; Wang et al., 2019). Leaf plasticity is an important index for measuring the ability of plants to adapt to heterogeneous environments, and leaf plasticity is often closely related to the high potential adaptability of plants to the environment (Liu et al., 2016; Rogowski et al., 2019). Soluble sugar (SS) is the product of plant photosynthesis. The SS content can reflect not only the intensity of plant photosynthesis but also plant nutrient metabolism and accumulation to a certain extent. Light exerts a certain effect on the accumulation of free radicals and reactive oxygen species in plants (Li et al., 2017). Therefore, studying the changes in physiological and biochemical characteristics and morphological structures of plants under shading conditions can reflect the response of a certain plant to light intensity and provide a deep understanding of its tolerance to shaded environments. In addition, such a study would be important for guiding agricultural and forestry production, improving the seeding survival rate and preservation rate, and improving production management measures.

DNA is the carrier of genetic information, and the real bearer of cell biological function is protein. In recent years, *Suaeda corniculata*, *Zingiber officinale*, and *Zea mays* have been studied using proteomic techniques to determine the dynamic changes in the plant proteome in response to stress (Mastrobuoni et al., 2012; Pang et al., 2016; Gao et al., 2020; Lv et al., 2020). The proteome constitutes all the proteins expressed by organisms. Zhu et al. (2021) comprehensively analyzed the leaf physiology and chloroplast proteome of wheat seedlings under salt and osmotic stresses. Lv et al. (2020) studied the photochemistry and proteomics of ginger under drought and shaded conditions. The goals of this research were as follows: (1) to detect changes in physiological indexes such as SS contents, peroxidase (POD) and superoxide dismutase (SOD) activities and leaf blade structure under different shading intensities; (2) to screen differentially expressed proteins (DEPs) under different shading treatments and perform functional annotation;, and (3) to perform Gene Ontology (GO) and Kyoto Encyclopedia of Genes and Genomes (KEGG) metabolic pathway enrichment analyses to elucidate the blueberry leaf photosynthetic system and differences in flavonoid accumulation and metabolism and determine the effect of shading on blueberry seedling growth mechanisms. This study will provide technical support and a theoretical reference for the planting, propagation and subsequent field treatments (summer rescue and protection) of blueberry seedling.

## Materials and Methods

### Plant Materials

Two-year-old potted “Biloxi” (Sharpblue × US329) southern highbush blueberry plants with upright and strong tree growth, strong yield, small pedicel marks, and fruit flavor were subjected to artificial shade for 2 months under environments with different gradients of light. The “Biloxi” cultivar experimental materials were obtained from the blueberry resource garden of Lishui White Horse Research Base (119°03′E, 31°35′N), Nanjing, Institute of Botany, Jiangsu Province and Chinese Academy of Sciences. The region is located in the transitional zone between north subtropical and subtropical monsoon climates and has four distinct seasons. The climate is mild and humid and has sufficient illumination and equal periods of rain and heat. The annual average temperature is 16°C, the annual average relative humidity is 77%, the annual average precipitation is 1,147 mm, the annual average sunshine is 1,969 h, and the annual average frost-free period is 224 d. The plum rain season is from late June to early July.

### Experimental Design

The experimental design was a single factor experiment. Three shading treatments were stablished, and different layers of black shading nets were used to control the light density (Wu et al., 2022). The three treatments were the following: no shading (CK, total light), 50% shading (T1, single-layer black shade net) and 80% shading (T2, two-layer black shade net). The light environment parameter is approximately 66,000 lx of total light intensity (minimum approximately 35,000 lx; maximum approximately 88,000 lx). Three replicates of each treatment were included, and each replicate consisted of 3–4 seedlings. The shading treatment test period was from the end of July 2020 to the end of September 2020. CK, T1 and T2 “Biloxi” leaves were collected every 15 days for the measurement of physiological and biochemical indexes, including chlorophyll soil and plant analyzer development (SPAD) values, SS contents, malondialdehyde (MDA) contents, POD activity, catalase (CAT) activity, and SOD activity. The changes in the cell structure and stomata in “Biloxi” leaves were observed by scanning electron microscopy (SEM) at the initial and final stages of different treatments. In addition, the protein and metabolite levels of “Biloxi” leaves in the last stage were further studied. Three biological replicates of each treatment were included to measure for all the indicators.

### Determination of Growth, Physiological and Biochemical Indexes

At the early stage in July and the last stage in September, the seedling height, ground diameter, and crown width of blueberry “Biloxi” were measured. Plant height: The distance from the base of the blueberry plant to the top was measured using a tape measure. Ground diameter: A Vernier caliper was used to measure the thickness of the plant’s main stem parallel to the base. Crown width: The width of the largest crown from east to west and north to south of the blueberry plant was measured by a tape measure. The SPAD values were then measured using a portable chlorophyll meter (SPAD-502, Minolta Camera Co., Osaka, Japan). Fifteen leaves of each treatment were randomly selected for the measurements to avoid the main vein of the leaf blade. The SS and MDA contents of blueberry “Biloxi” leaves were determined using a plant soluble sugar (s-sugar) ELISA kit and plant malondialdehyde (MDA) ELISA kit (Shanghai Yingxin Laboratory Equipment Co., Ltd., Shanghai, China). The POD, SOD, and CAT activities of blueberry leaves were measured by plant peroxidase (POD)/superoxidase dismutase (SOD)/catalase (CAT) ELISA in units of ng/g FW. The ribulose 1,5-bisphosphate carboxylase/oxygenase (rubisco) activity was measured using the rubisco assay kit (A151-1-1, Nanjing jiancheng Bioengineering Institute, China).

### Micromorphological Observation of Blueberry Leaf Epidermis by Scanning Electron Microscopy

Fresh leaves from the different shading treatments were compared by SEM. A square section of approximately 0.5 × 0.5 cm was obtained by cutting the leaves, avoiding the main veins. The leaves were then fixed with 2.5% (v/v) glutaraldehyde and stored at 4°C until use. The leaves were then freeze-dried and gold-plated according to Zhang et al. (2017). SEM observations were performed according to the FEI Quanta 200E SEM basic instructions. Data on the leaf epidermal features were obtained using XT microscope server software (Zhang et al., 2017).

### Sample Preparation for Tandem Mass Tag-Labeled Quantitative Proteomics

Blueberry leaves from the CK, T1 and T2 groups (named CK-1, CK-2, CK-3, T1-1, T1-2, T1-3, T2-1, T2-2, and T2-3) were collected for Tandem Mass Tag (TMT)-labeled quantitative proteomics analysis. The basic procedure of the TMT-labeled quantitative proteomics experiment was as follows: the total protein in the CK-1, CK-2, CK-3, T1-1, T1-2, T1-3, T2-1, T2-2, and T2-3 samples was extracted separately, and part of the protein was extracted for determination of the BCA protein concentrations (Smith et al., 1985). Then, 10 μg of protein was obtained from each sample and detected using a 12% SDS–PAGE gel. The isolated gels were stained with Coomassie Bright Blue using an eStain LG protein staining apparatus. The stained gels were imaged using the Automatic Digital Gel Image Analysis System (Supplementary Figure 1). In addition, according to the measured protein concentrations, 50 μg of protein was collected from each sample, and the blueberry samples from different groups were diluted to the same concentration and volume for trypsin enzymatic hydrolysis and peptide TMT labeling with lysate. The same amount of labeled sample was then mixed and separated by reversed-phase chromatography. Liquid chromatography (LC) was performed using an Agilent 1,100 HPLC with a 2.1 × 150 mm, 5 μm Agilent Zorbax Expense-C18 narrow-diameter column with the following parameters: detection wavelength, UV210 nm and 280 nm; mobile phase A, ACN-H2O (2:98, V/V), ammonia water was used to adjust the pH to 10; mobile phase B, ACN-H2O (90:10, v/v), ammonia water was used to adjust the pH to 10; and flow rate, 300 μL/min. Finally, the samples were tested by LC–MS/MS; the sample was loaded into an Acclaim Pepmap 100 100 μm × 2 cm precolumn (RP-C18, Thermo Fisher) at a flow rate of 300 nL/min and then separated by an Acclaim Pepmap RSLC 75 μm × 50 cm analytical column (RP-C18, Thermo Fisher). The mass resolution of the first-stage MS was set to 120,000, the automatic gain control value was set to 3e6, and the maximum injection time was 80 MS. The mass spectrometry was set to the full scanning charge/mass ratio m/z range of 300–1,650, and the 15 peaks were scanned by MS/MS. All MS/MS chromatograms were collected using high-energy collision lysis in the data-dependent positive ion mode, and the collision energy was set to 32. The resolution of MS/MS was set to 45,000, the automatic gain control was set to 1e5, and the maximum ion injection time was 80 MS. The dynamic elimination time was set to 30 s. After the final database search, the proteins were qualitatively and quantitatively analyzed, and Proteome Discover 2.4 (Thermo Fisher Company) data were used for analysis.

### Total RNA Extraction and Quantitative Real-Time Fluorescent Polymerase Chain Reaction (RT–qPCR) Analysis

Total RNA from blueberry leaves was extracted using the polysaccharide and polyphenol total isolation kit (spin column) (BioTeke, Wuxi, China), and total RNA was transcribed to cDNA using PrimeScript RT Master Mix (Perfect Real Time) (Takara, Dalian, China). Gene transcripts were detected using TB Green Premix Ex Taq II (Tli RNaseH Plus) (Takara, Dalian, China) with an ABI Viia7 real-time PCR system (Applied Biosystems, United States) according to the manufacturer’s protocols. Actin (forward primer: 5-AGGCTAACCGTGAGAAGATGAC-3; reverse primer: 5-AGAGTCCAGCACGATTCCAG-3) was used as the reference gene (Supplementary Table 1; Zifkin et al., 2012). The relative expression level was calculated using the 2^–ΔΔ*Ct*^ method.

### Combined Analysis of the Proteome and Metabolome

For the specific parameters of the metabolite extraction process, multivariate statistical methods and differentially expressed metabolite (DEM) enrichment of the nine blueberry leaf samples, please refer to previous studies in our laboratory (Wu et al., 2022). The top 20 DEPs and DEMs were used to calculate the expression correlation between DEPs and DEMs by the Pearson correlation algorithm using relative content data. The DEPs and DEMs were analyzed by mapping to the KEGG pathway database to obtain their common pathway information. The KGML file is a sub database in the KEGG database that contains not only the relationship of graphic objects in the KEGG pathway but also information of direct homologous genes in the KEGG gene database. Based on this analysis, we obtained the network relationship between DEPs and DEMs through KGML network analysis, and more systematically studied the interaction between the proteome and metabolome.

### Statistical Analysis

SPSS 22.0 (SPSS Institute Inc.) was used to perform analyses of variation (ANOVAs) of the physiological parameter data. The results for each parameter are presented as the means from three biological replicates. The significance level of the differences among samples was determined by Duncan’s multiple tests at the 0.05 probability level.

## Results

### Effects of Different Shading Treatments on Blueberry Leaf Physiology

**FIGURE 1:**
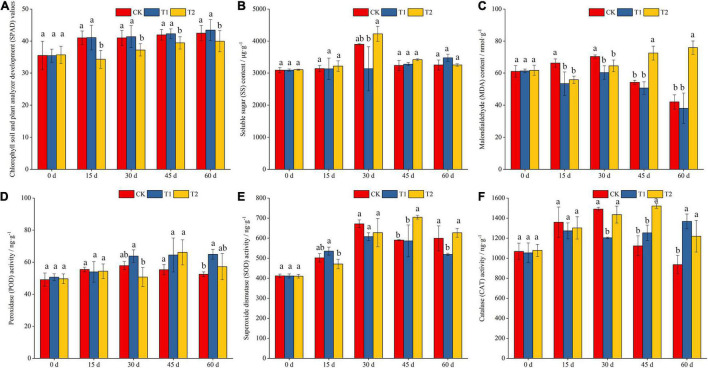
Effects of different shading treatments on the physiological indexes of blueberry leaves. Chlorophyll soil and plant analyzer development (SPAD) values in different shading treatment **(A)**; soluble sugar (SS) and malondialdehyde (MDA) contents in different shading treatment **(B,C)**; activities of peroxidase (POD), superoxide dismutase (SOD) and catalase (CAT) in different shading treatment **(D–F)**.

The SPAD value in the leaves of blueberry seedlings increased with extension of the shading duration. With the exception of the results for the results for the initial shading period (0 d), no significant difference was found among all the treatments, and the SPAD value of blueberry leaves under the CK and T1 treatments was significantly higher than that under the T2 treatment at other periods (Figure 1A). On the 30th day of shading treatment, the content of SS in the T2 treatment was significantly higher than that in the T1 treatment, and the content of SS in the CK group was also significantly higher than that in the T1 group (Figure 1B). The MDA content showed different trends with shading time in this study. The MDA content in the CK group was significantly higher than that in the T1 and T2 groups after 15 and 30 days of shading, respectively. The MDA content in the T2 group was significantly higher than that in the CK and T1 groups at 45 and 60 days, respectively (Figure 1C).

SOD and POD are two important protective enzymes in the enzymatic defense system of cells that resist damage from reactive oxygen species. These enzymes act as scavengers of superoxide anion radicals (O_2_^⋅–^) and hydroxyl radicals (⋅OH), respectively. The changes in SOD activity in blueberry leaves generally showed an increasing trend with extension of the shading duration (Figure 1D). However, POD activity generally increased first and then decreased with extension of the shading duration (Figure 1E). The results indicated that the seedlings could eliminate the reactive oxygen and hydroxyl free radicals produced by stress by increasing the activities of SOD and POD early during the shading period and maintaining the physiological balance in the cells. In addition, CAT activity in blueberry leaves showed different trends under the different shading treatments (Figure 1F). The CAT activity in the CK and T2 treatments generally increased first and then decreased with extension of the shading duration. The activity of CAT under the CK and T2 treatments was significantly higher than that under the T1 treatment on the 30th day of the shading treatment, and the activity of CAT under the T2 treatment was significantly higher than that under the CK treatment after 45 and 60 days of shading.

### Effects of Different Shading Treatments on the Growth of Blueberry

After 60 days shading, growth indexes showed that shading affects blueberry growth (Table 1). Compared with the results before shading, the T1 treatment yielded the largest increases in the crown width and ground diameter (12.08 and 2.43 cm), and the values obtained for the T1 group were significantly higher than those of the CK and T2 groups. The T1 treatment promoted the growth of blueberries, and the average increases in plant height, crown width and ground diameter obtained with this treatment were 58.50, 24.54, and 23.35% higher than the values of the CK group, respectively. The growth of blueberry was significantly inhibited by the T2 treatment, and the average increases in plant height, crown width, and ground diameter obtained with this treatment were lower than the values of the CK group. Moreover, the rubisco activity of blueberry decreased with the increase of shading time and intensity (Table 1). Therefore, moderate shading during this period is beneficial to the growth of blueberry seedlings.

**TABLE 1:** Changes in growth indexes and rubisco activity of blueberry “Biloxi” under different shading treatments.

Treatment	Plant height increment/cm	Crown width increment/cm	Ground diameter increment/cm	Rubisco activity/nmol⋅min^–1^⋅g^–1^
CK	14.70 ± 5.01a	9.70 ± 2.60b	1.97 ± 0.54b	–7.35 ± 2.59a
T1	23.30 ± 5.85a	12.08 ± 3.39a	2.43 ± 0.37a	–5.10 ± 2.07a
T2	7.80 ± 2.94b	6.91 ± 1.80c	1.42 ± 0.40c	–12.34 ± 2.72b

*The data are shown as the means ± standard deviations. Lowercase letters indicate significant differences between different treatments (P < 0.05).*

**FIGURE 2:**
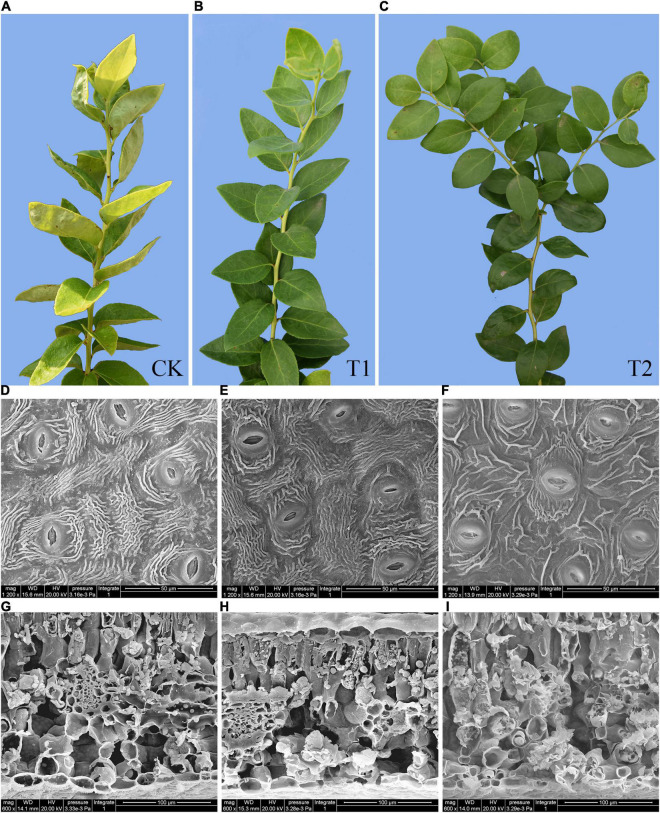
The appearance and scanning electron microscopy (SEM) observations of blueberry leaves. The appearance of fresh blueberry leaves under different shade treatments **(A–C)**. The changes in the lower epidermis **(D–F)** and transections **(G–I)** of young blueberry leaves under different shade treatments were observed by SEM.

After 60 days of shading treatment, the appearance of fresh blueberry leaves showed difference. The blueberry leaves of the T1 group were greener than those of the CK group (Figures 2A,B). The leaves in the T1 and T2 treatment groups may not curl due to the absence of strong light damage (Figures 2B,C). Moreover, we observed the changes and differences in the stomatal distribution and cross-sectional structure of blueberry leaves under different shading intensities by SEM. The distribution of stomata in leaves was observed by 1,200 × SEM, and the results showed that the stomatal apertures of leaves were long and ovate, were scattered randomly on the back of leaves, showed an irregular orientation (Figures 2D–F). After shading treatment, some differences in stomatal opening and other traits were detected among the treatments. Under the T1 treatment, the stomatal opening was large or small (Figure 2E), whereas under the T2 treatment, the stomatal morphology lost elasticity and showed signs of decay, and the leaf veins were fewer or shallower (Figure 2F). By observing the cross-sectional structure of leaves, little difference were found between the CK and T1 treatments, and palisade, vascular bundle and spongy tissues could be clearly observed (Figures 2G,H). Under the T2 treatment, the cells dissolved, the morphology was incomplete, the vascular bundles disappeared, and no supporting skeleton was detected showed that (Figure 2I).

### Proteomic Analysis of Blueberry Leaves

A proteome analysis of blueberry leaves after 60 days of shading treatment was conducted to further reveal the effects of shading on their growth and related metabolite synthesis. Taking OD_562_ as the abscissa and the standard protein concentration as the ordinate, the standard curve and regression equation are shown in Supplementary Figure 1A; for the equation, *R*^2^ = 0.99, indicating that the standard curve had a high degree of fit. The absorbance values of the nine blueberry leaves tested can be substituted into the regression equation to calculate the concentration (CK-1 = 4.56 μg/μL; CK-2 = 1.93 μg/μL; CK-3 = 2.10 μg/μL; T1-1 = 2.21 μg/μL; T1-2 = 2.26 μg/μL; T1-3 = 2.96 μg/μL; T2-1 = 2.57 μg/μL; T2-2 = 1.38 μg/μL; T2-3 = 3.32 μg/μL). The SDS–PAGE results are shown in Supplementary Figure 1B. The samples were detected by LC–MS/MS, and the library was searched. The results of qualitative and quantitative analysis using the criteria for proteins (false discovery rate < 0.01) identified 4,883 protein groups, 18,224 peptides, and 41,996 effective spectra.

**FIGURE 3:**
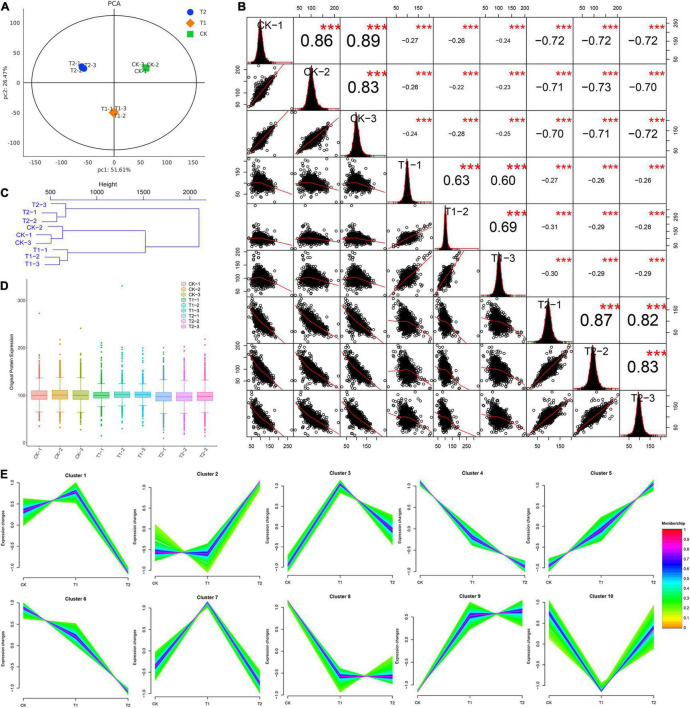
Reliable protein analysis and data quality control of nine blueberry samples. **(A)** Principal component analysis, **(B)** correlation analysis of nine blueberry samples. In the upper triangle (upper right of diagonal), the number represents the correlation value of the two samples; the asterisks * indicate the significance level (***P < 0.001). The lower triangle (lower left of the diagonal) shows the scatter plot of the two samples. The red curve shows the fitting trend. A larger slope indicates a stronger correlation between the two samples. The diagonal line shows the distribution of the self-expression of the sample. **(C)** Hierarchical cluster analysis. Each branch end represents a sample. **(D)** Box plot of credible protein expression. The x-axis shows the sample name, and the y-axis shows the sample expression. **(E)** Cluster analysis of expression trend of the credible proteins in blueberry leaves.

After the original data were retrieved from the database, the trusted proteins were then screened according to the criteria of Score SequestHT > 0 and unique peptide ≥ 1, and blank values were removed. A principal component analysis (PCA) was performed by using the expression levels of trusted proteins. Each point represents a repeat, and different groups are distinguished by different colors. The differences between samples in the same group were small, whereas significant differences between different groups were found (Figure 3A). In addition, the correlation analysis of nine samples of known proteins showed that the correlation of the samples within a group was strong and that the reproducibility was good. The correlation between groups was weak (Figure 3B). Using reliable quantitative expression data, the Euclidean distance between the nine blueberry leaves was calculated, and the sample distance matrix was then subjected to hierarchical cluster analysis. The samples gathered in the same branch exhibited similar expression and characteristics. A hierarchical clustering of the Euclidean distances revealed that the difference in proteins within the same group was small. A hierarchical clustering dendrogram of sample Euclidean distances showed that the quantitative protein expression data in the T2 group were significantly different from those in the T1 and CK groups (Figure 3C). A boxplot analysis of known protein expression revealed that the degree of data fluctuation in the same treatment group was similar, and the data in the T1 group were relatively concentrated (Figure 3D). An expression tend cluster analysis was performed using credible proteins, and 10 groups of cluster groups (protein groups) with different dynamic patterns were obtained (Figure 3E). The proteins in each cluster group exhibited similar expression characteristics. The dynamic patterns of proteins in different cluster groups were significantly different. In addition, 32 transcription factor family members were distributed in blueberry leaves, and WRKY transcription factor family members were the most abundant (Supplementary Figure 2).

### Differentially Expressed Proteins in Blueberry Leaves

**FIGURE 4:**
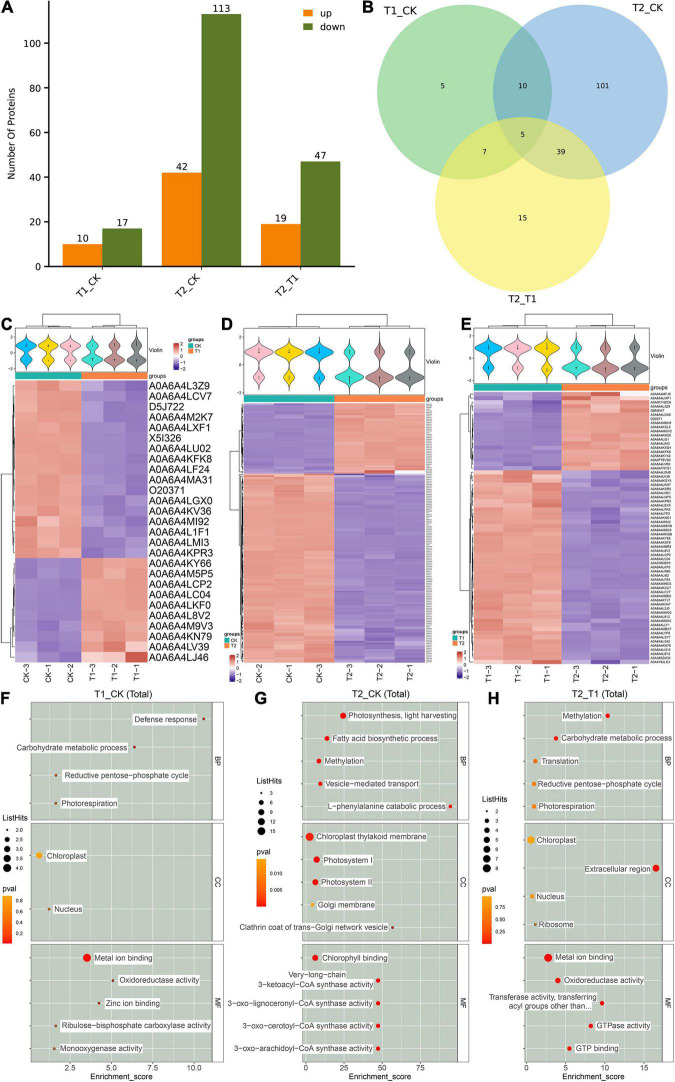
Analysis of differentially expressed proteins (DEPs) between different groups. The number of DEPs in different shading treatment groups **(A,B)**. The violin diagram and heat map of DEPs between different treatment groups **(C–E)**. GO enrichment score map of DEPs between different groups **(F–H)**.

Based on known proteins, two standards were selected to calculate the differences among the three groups of blueberry samples (fold change = 2 times and *p*-value < 0.05). The same type of samples can appear in the same cluster by clustering. The results showed that 27 DEPs were found between the CK and T1 groups, including 10 DEPs that were significantly upregulated in the T1 group (Figures 4A,C). One hundred fifty-five DEPs were found between the CK and T1 groups, and these included 113 DEPs that were significantly downregulated in the T2 group compared with the CK group (Figures 4A,D). Nineteen upregulated and 47 downregulated DEPs were found in the T2 group compared with the CK group (Figures 4A,E). Venn diagrams were used to analyze the characteristics and commonalities of DEPs in each group and revealed five DEPs (A0A6A4LCP2, A0A6A4KY66, A0A6A4KPR3, A0A6A4LCV7, and A0A6A4LC04) that overlapped among the three groups (Figure 4B). The violin diagram is a combination of a box line diagram and a density diagram. A flatter violin box indicates that the data are more concentrated. The outline of the violin box reflects the probability distribution of the expression value. Different color fills represent different samples. The cluster heatmap below the violin was clustered according to protein expression: the red color reflects highly expressed proteins, and the blue color represents weakly expressed proteins (Figures 4C–E).

A GO enrichment analysis of the obtained DEPs was performed, and differences in the enriched GO items were found in the different comparison groups (Supplementary Figure 3). Metal ion blinding (A0A6A4LU02, A0A6A4KPR3, A0A6A4KFK8, and A0A6A4MI92) and chloroplasts (O20371, D5J722, and A0A6A4KFK8) were the most abundant in the comparison between the T1 and CK groups (Figure 4F). In the comparison between the T2 and CK groups, metal ion binding (17 DEPs), chloroplast thylakoid membrane (16 DEPs) and photosystem I (10 DEPs) were the most abundant (Figure 4G). Moreover, metal ion binding (8 DEPs) and chloroplasts (7 DEPs) were the most enriched in the comparison between the T2 and T1 groups (Figure 4H).

**FIGURE 5:**
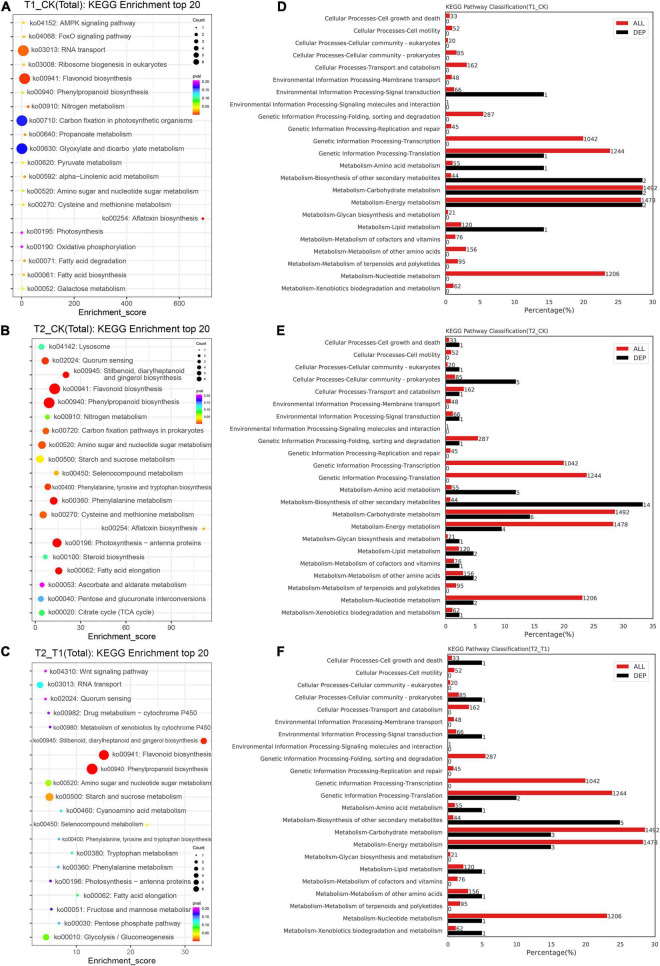
Top 20 KEGG pathways and KEGG pathway classification of differentially expressed proteins. The KEGG enrichment top 20 pathways **(A–C)**. The percentage and number of KEGG pathway classification between different treatment groups **(D–F)**.

To further understand the DEP functions under the different shading treatments, a KEGG analysis was conducted. KEGG is a major public database for the systematic analysis of protein metabolism pathways in cells. The KEGG annotation analysis showed that the T1 group exhibited a higher abundance of proteins involved in flavonoid biosynthesis (ko00941) compared with the CK group (Figure 5A). The proteins showing differential expression between the T2 and CK groups were most highly enriched in flavonoid biosynthesis (ko00941), phenylpropanoid biosynthesis (ko00940) and photosynthesis-antenna (ko00196) (Figure 5B). In addition, phenylpropanoid biosynthesis (ko00940) and flavonoid biosynthesis (ko00941) were also the most highly enriched in the proteins showing differential expression between the T2 and T1 groups (Figure 5C). The comparison of the distribution of DEPs and species proteins at KEGG level 2 showed that metabolism-biosynthesis of other secondary metabolites, carbohydrate metabolism and energy metabolism included two DEPs (Figure 5D). Interestingly, metabolism-biosynthesis of other secondary metabolism pathways had 14 DEPs between the T2 and CK groups (Figure 5E) and five DEPs between the T2 and T1 groups (Figure 5F).

### Relative Expression Analysis Validation

Five DEPs (A0A6A4LCP2, A0A6A4KY66, A0A6A4KPR3, A0A6A4LCV7, and A0A6A4LC04) overlapping across the three groups and four important DEPs were selected to verify the accuracy of the DEP results by RT–qPCR analysis. The results showed that the expression of the genes corresponding to the selected DEPs was approximately 50% consistent with the results from the proteomics analysis (Supplementary Table 2). The RT–qPCR results reflected the changes in mRNA levels, and the changes in transcription levels are not necessarily consistent with the changes in protein levels.

### Differentially Expressed Protein Interaction Network Analysis

**FIGURE 6:**
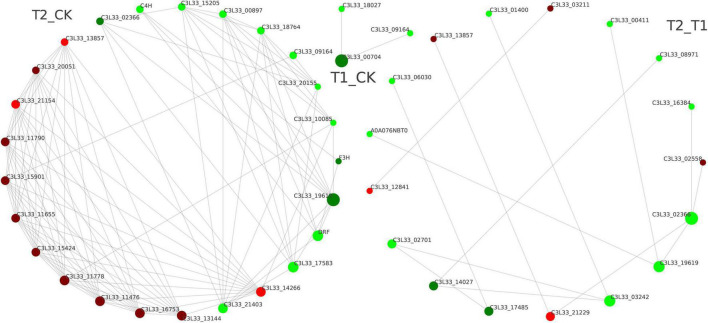
Protein–protein interaction maps of differentially expressed proteins.

The top 25 DEPs with connectivity were selected, and the interaction network was drawn using the STRING database (Figure 6). The circle in Figure 6 represents the DEPs, the red color represents the upregulated proteins, and the green color represents the downregulated proteins (Figure 6). In the T1 and CK groups, an interaction was only found between a downregulated DEP (C3L33_00704) and the other two downregulated DEPs (C3L33_18027 and C3L33_09164). Between the CK and T2 groups, the proteins trans-cinnamate 4-monooxygenase (C4H, CYP73A), naringenin 3-dioxygenase (F3H) and bifunctional dihydroflavonol 4-reductase/flavanone 4-reductase (DFR), which are related to flavonoid synthesis were closely related to other DEPs. Interestingly, C4H, F3H, and DFR also exhibit high connectivity and receive mutual regulation. These results suggest that these DEPs may interact with each other to promote the expression of related genes and the accumulation of key metabolites.

### Analysis of Differentially Expressed Metabolites Involved in the Flavonoid Biosynthesis Pathway

**FIGURE 7:**
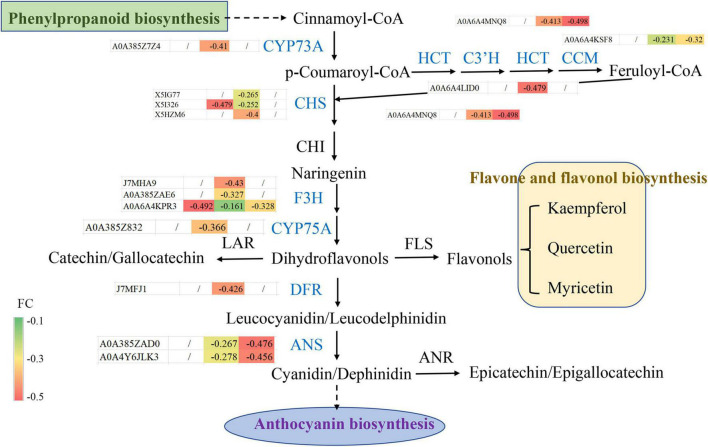
Protein expression patterns of flavonoid biosynthesis pathway members in blueberry. The fold changes (between −0.1 and −0.5) of differentially expressed proteins among different groups, including T1 vs. CK, T2 vs. CK and T2 vs. T1, are shown.

To further understand the molecular changes in the regulated proteins involved in flavonoid biosynthesis, the protein expression patterns of flavonoid biosynthesis pathway members in blueberry under different shading treatments were analyzed (Figure 7). Among the DEPs, 14 were involved in the flavonoid biosynthesis pathway; for example, C4H catalyzes the hydroxylation of trans-cinnamic acid to *p*-coumaric acid and belongs to the CYP73 family of cytochromes P450 (CYP450). The C4H DEP A0A385Z7Z4 may include the most common catalytic enzymes involved in the diverse metabolite biosynthesis between the CK and T2 groups. Chalcone synthase (CHS) regulates a crucial reaction in the flavonoid pathway, isomerizing naringenin chalcone by catalyzing the condensation of 4-coumaroyl-CoA and malonyl-CoA. The results indicated that three DEPs (X5IG77, X5I326, and X5HZM6) showed a decrease in expression in blueberry leaves under shading treatment. Chalcone isomerase (CHI) produces naringenin, and the pathway then diverges into side branches with different classes of flavonoids. The downstream candidate catalytic enzyme DEPs J7MHA9, A0A385ZAE6, A0A6A4KPR3, A0A385Z832, J7MFJ1, A0A385ZAD0, and A0A4Y6JLK3 were annotated as F3H, flavonoid 3′,5′-hydroxylase (F3′,5′H, CYP75A), DFR and anthocyanidin synthase (ANS) in blueberry. The above-described results show that DEPs contribute to the main flavonoid biosynthesis pathway, and the joint regulation of DEPs *via* upstream and downstream flavonoid biosynthesis pathways may result in changes in flavonoid levels.

### Combined Analysis of Differentially Expressed Proteins and Differentially Expressed Metabolites

**FIGURE 8:**
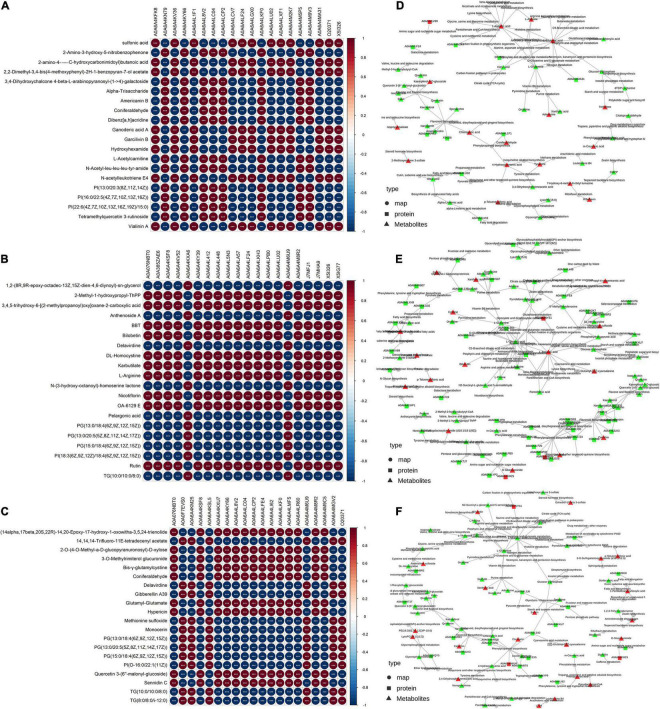
Correlation analysis of differentially expressed proteins and differentially expressed metabolites and KGML network diagram. **(A–C)** The asterisks (***) represent that the significance of the correlation *p*-value was less than 0.001, respectively. The T1 vs. CK, T2 vs. CK and T2 vs. T1 are shown. **(D–F)** The squares represent proteins, and the triangles represent metabolites. The red indicates upregulated protein or metabolites, and the bright green color indicates downregulated protein or metabolites.

In the correlation analysis between the top 20 DEPs and DEMs, each row consists of different DEMs, and each column is a DEP. The red color indicates a significant positive correlation, and the blue color indicates a significant negative correlation. A deeper color intensity indicates a greater correlation and a smaller circle diameter indicates that the correlation is close to zero (Figures 8A–C). The correlation between the expression of DEPs and DEMs differed among the different comparison groups. In addition, through the network relationship between proteins and metabolites, we can more systematically study the interaction between the proteome and metabolome among different comparison groups. Three DEMs (L-aspartic, L-arginine and glutamic acid) interacted with multiple proteins or maps, whereas many network interactions between the significantly upregulated metabolites kaempferol-3-O-glucoside, chlorogenic acid, coniferaldehyde, 4-hydroxycinnamic acid, and tyramine and downregulated DEPs or DEMs were found between the T1 and CK groups (Figure 8D). Interestingly, compared with the other comparison groups, the interaction network between DEPs and DEMs between the T2 and CK groups was more abundant (Figure 8E). The KGML network showed that upregulated catechin exhibited an interaction network with other flavonoid biosynthesis or flavone and flavonol biosynthesis, and most of the metabolites related to flavonoid synthesis, such as quercitrin, rutin, isoquercitrin, kaempferol, and quercetin, were differentially downregulated (Figure 8F).

## Discussion

### Morphological Parameters and Phenotypic Changes of Blueberry Leaves

Light plays an important role in controlling plant morphogenesis, metabolism, growth and development (Cheynier et al., 2013). Light is one of the most important and direct external factors affecting plant growth, development, and secondary metabolism (Hussain et al., 2019a,b). In response to different light intensities, many plants undergo physiological, biochemical, and morphological changes (Zoratti et al., 2014). Different shading treatments exerted significant effects on the morphology of blueberry seedlings, which directly affected the external morphology, growth and development of individual plants (Figure 2). Morphological changes in leaves, which are the main organs of photosynthesis and transpiration, can reflect the stress resistance of plants to a certain extent (Lv et al., 2020). Stomata are the channels for transpiration and photosynthetic substances to enter cells, and their behaviors are closely related to the transpiration and photosynthetic performance of plant leaves (Zhang et al., 2017). Under the T2 treatment, stomatal apertures lost elasticity (Figure 2E), leaf cells appeared dissolved in cross-section, the morphology was incomplete, vascular bundles disappeared, and no supporting skeleton was observed (Figure 2I), which indicated that excessive shading stress affected the normal growth and development of blueberry leaves. In this study, the T1 treatment yielded the highest and significant increases in the crown width and ground diameter of blueberry (Table 1), which showed that blueberry seedlings need moderate shading in summer. A shading degree of 50% (T1 treatment) resulted in the best growth, and too much shading (80%, T2 treatment) was not conducive to the growth of blueberry.

### Physiological Parameters of Blueberry Leaves

Chlorophyll in plant leaves is the main substance for plants to absorb light energy during photosynthesis (Lv et al., 2020). The chlorophyll content can reflect the photosynthetic capacity of leaves. Most plants synthesize a large amount of chlorophyll under weak/low light to capture more light energy, but there are differences between different plants (Lv et al., 2020). In summary, the chlorophyll content is closely related to photosynthesis, which can reflect the ability of plants to produce assimilates to some extent (Ii and Webber, 2005). The results from this study showed that shading increased the SPAD value of blueberry leaves, whereas severe shading significantly reduced the SPAD value (Figure 1A). This finding differs from the results reported by Lv et al. (2020). The reason for this phenomenon in our research may be that blueberry leaves under heavy shading appear yellow-green in color. Herein, the SS content of blueberry leaves after 30 days of the CK and T2 treatments was significantly higher than that obtained with the T1 treatment (Figure 1B). This finding shows that SS accumulated continuously during plant growth and was highly accumulated after 30 days of treatment, resulting in the highest SS content at this time. SS is a type of osmotic regulatory substance in plants. The change on its content will affect the osmotic potential of plants, which is conducive to maintaining the plant water and nutrition balance under adversity and improving plant resistance to stress (Dennis et al., 2020). SOD is the first line of defense in the plant antioxidant protection system, which mainly removes O_2_^⋅–^ in plants. This protective enzyme can prevent the damage caused by oxygen free radicals to the cell membrane. POD is a protective enzyme that can protect cells from reactive oxygen species-induced damage. In this study, the changes in SOD and POD activities in blueberry leaves were basically the same, showing a trend of first increasing and then decreasing (Figures 1D,E). This result indicated that blueberry seedlings at the early stage of shading could maintain the physiological balance of cells by increasing the activities of SOD and POD and removing reactive oxygen species and hydroxyl radicals produced by stress. At the later stage of the T2 shading treatment, the MDA content in blueberry leaves increased significantly (Figure 1C), indicating that the accumulation of MDA was the result of reactive oxygen species. It was also found that rubisco activity decreased with the increase of shading time and intensity, which was similar to the results of Hussain et al. (2019a). This may be shading inhibited the expression and synthesis of rubisco enzyme, resulting in the decrease of rubisco activity.

### Photosynthesis and Flavonoid-Related Proteins in Blueberry

The proteome refers to all the proteins expressed by organisms. To explore its functional regulation at the protein level, we can analyze the dynamic changes of proteins in cells and the expression levels of proteins from a holistic perspective using corresponding means to understand the interactions and relationships between proteins and reveal the role of proteins in cell life activities (Lv et al., 2020). The application of proteomics can provide a new theoretical perspective and solutions for elucidating the mechanisms of the plant stress response. In recent years, high-throughput proteomics technology has been used to analyze the dynamic changes in the proteome of *Malus halliana* (Jia et al., 2019), *Zingiber officinale* (Lv et al., 2020), wheat (Zhu et al., 2021) and other plants during stress responses. Because proteins are the ultimate executors of cell function, a series of physiological and biochemical reactions of cells to environmental signals are mostly related to the interaction between proteins and changes in protein activity. Herein, the DEPs related to the photosystem and chloroplasts were significantly enriched through GO enrichment analysis (Figures 4F–H), and the results were similar to those reported by Lv et al. (2020). The KEGG analysis performed in the study showed that the phenylpropanoid and flavonoid biosynthesis pathways were the most enriched (Figures 5A–C). Flavonoids play an important role in plant resistance to environmental stress. Similien et al. (2016) found that the flavonoid yield in American Skullcap was 25% higher under shade. The DEP interaction network analysis showed that C4H, F3H, and DFR exhibit high connectivity and receive mutual regulation (Figure 6). This finding indicates that C4H and F3H in the early flavonoid biosynthesis pathway and DFR in the downstream step of the flavonoid pathway are closely related to flavonoid synthesis and accumulation. C4H acts as a rate-limiting enzyme in phenylpropanoid biosynthesis and exhibits the same markedly reduced expression levels in the shading treatment group, which may lead to differential accumulation of *p*-coumaroyl-CoA upstream of flavonoid biosynthesis. In this study, 14 DEPs involved in flavonoid biosynthesis, including CHS, F3H, F3′,5′H, and ANS, were significantly downregulated during the shading period (Figure 7), which was similar to the results reported by Xu et al. (2020). Previous studies have suggested that an inactivating mutation of CHS can lead to decreased flavonoid accumulation in plants (Li et al., 1993). CHS (Li et al., 1993) and ANS (Shimada et al., 2005) are key enzymes in flavonoid biosynthesis. The protein expression levels of CHS and ANS under shading treatment were lower than those in the CK group (Figure 7), which indicated that more light is conducive to the synthesis of CHS and ANS proteins. In addition, a higher shading intensity affects not only plant growth but also the accumulation of flavonoids and other secondary metabolites (Semchenko et al., 2012). Bergquist et al. (2007) found significant differences in flavonoid concentrations (up to 100%) between shaded and unshaded *Spinacia oleracea* at different times of a season, which indicated that the use of shading nets can be used to produce *Spinacia* in terms of the flavonoid concentration and composition. Therefore, understanding the interactions and relationships among proteins under different shading intensities can further reveal the growth dynamics of plants under different treatments as well as the regulation of photosynthesis and secondary metabolite accumulation.

## Conclusion

Compared with the CK and T2 shading treatments, the T1 treatment yielded the largest increases in plant height, crown width, and ground diameter of blueberry, which indicated that moderate shading in summer is beneficial to blueberry growth. A proteomics revealed five DEPs, namely, A0A6A4LCP2, A0A6A4KY66, A0A6A4KPR3, A0A6A4LCV7, and A0A6A4LC04, among the three groups. KEGG annotation showed that the DEPs were most enriched in the phenylpropanoid and flavonoid biosynthesis pathways. Moreover, the DEP interaction network analysis revealed that C4H, F3H, and DFR have high connectivity and receive mutual regulation. These results suggest that these DEPs may interact with each other to promote the expression of related genes and the accumulation of key metabolites. In conclusion, 50% shading is helpful for blueberry growth in a hot summer season.

## Data Availability Statement

The original contributions presented in the study are included in the article/Supplementary Material, further inquiries can be directed to the corresponding author/s.

## Author Contributions

YW and WW designed the study. YW, CZ, and CS performed the experiments. YW, ZH, and LL analyzed the data. YW wrote the manuscript. WL and WW revised the manuscript. All authors have read and agreed to the published version of the manuscript.

## Conflict of Interest

The authors declare that the research was conducted in the absence of any commercial or financial relationships that could be construed as a potential conflict of interest.

## Publisher’s Note

All claims expressed in this article are solely those of the authors and do not necessarily represent those of their affiliated organizations, or those of the publisher, the editors and the reviewers. Any product that may be evaluated in this article, or claim that may be made by its manufacturer, is not guaranteed or endorsed by the publisher.

## Supplementary Material

Supplementary Figure 1 Protein quantitative standard curve **(A)** and SDS–PAGE analysis of samples **(B)**.

Supplementary Figure 2 Distribution of the transcription factor family in blueberry leaves.

Supplementary Figure 3 Comparison of the differentially expressed proteins and all blueberry proteins at Gene Ontology classification level 2.

## Abbreviations

SS: soluble sugar
POD: peroxidase
SOD: superoxide dismutase
GO: Gene Ontology
KEGG: Kyoto Encyclopedia of Genes and Genomes
SPAD: chlorophyll soil and plant analyzer development
MDA: malondialdehyde
CAT: catalase
SEM: scanning electron microscopy
TMT: tandem mass tag
LC: liquid chromatography
DEPs: differentially expressed proteins
RT–qPCR: quantitative real-time fluorescent polymerase chain reaction
C4H (CYP73A): trans-cinnamate 4-monooxygenase
F3H: naringenin 3-dioxygenase
DFR: bifunctional dihydroflavonol 4-reductase/flavanone 4-reductase
CYP450: cytochromes P450
ANS: anthocyanidin synthase
F3′,5′H (CYP75A): flavonoid 3′,5′-hydroxylase
CHI: chalcone isomerase
CHS: chalcone synthase
DEMs: differentially expressed metabolites.
