# Supplementary material for: Comparative Analysis of the Morphological, Physiological, Proteomic, and Metabolic Mechanisms of the “Biloxi” Blueberry Response to Shade Stress
Source: Front Plant Sci. 2022 May 3;13:877789. doi: 10.3389/fpls.2022.877789 (PMC9111170; doi:10.3389/fpls.2022.877789)
Supplement: Supplementary file 1 [file Data_Sheet_1.docx]

**Table S1.** Gene-specific primers used for RT–qPCR.

| Name | Forward primer | Reverse primer | Amplicon length (bp) |
| --- | --- | --- | --- |
| A0A6A4LCP2 | CCCAATGGTGGGTGCATCGG | CCTGCTCTTTCCCGGCCTTG | 97 |
| A0A6A4KY66 | GGTGTTGCCGGACAATGGG | TAAGTCCACTGGGCCTCGCA | 131 |
| A0A6A4KPR3 | CCCGACCTCACACTCGGACT | CGTCCACGGGCTGAACTGTG | 133 |
| A0A6A4LCV7 | TGTGGGTTGTGCTCGGAAGC | TCTGCCTCATCCCATCGGCAA | 148 |
| A0A6A4LC04 | AGAGCTTCAAGGCCGCACTG | GACGGCAAAGTCGCGGATGA | 125 |
| A0A6A4MA31 | CAGCTCTCGAGGTGGAGGTCA | GCCTTGGCGGGTAAACCGAT | 142 |
| A0A6A4LU02 | TGCAGCCCGGATGATTCTTGC | AGGAAGACGGGTTGCCAGGA | 139 |
| A0A385Z7Z4 | GGGTCGAGTTCGGGTCGAGA | TTCCTCCAGTGCTCGCCGTA | 100 |
| J7MFJ1 | GAGCAACCGTTCGCGATCCA | CGTTCAGGTCCGCCTTCCAC | 102 |
| Actin | AGGCTAACCGTGAGAAGATGAC | AGAGTCCAGCACGATTCCAG | 127 |

**Table S2.** Proteomic analysis and RT–qPCR assay of nine related key differentially expressed genes.

| Name | CK abundances | T1 abundances | T2 abundances | CK expression | T1 expression | T2 expression |
| --- | --- | --- | --- | --- | --- | --- |
| A0A6A4LCP2 | 89.3±2.88 | 197.03±3.37 | 13.7±3.37 | 1.00±0.09 | 13.00±4.45 | 5.10±1.06 |
| A0A6A4KY66 | 85.77±2.30 | 175±3.14 | 39.23±3.14 | 1.02±0.27 | 2.55±0.37 | 0.73±0.08 |
| A0A6A4KPR3 | 181.43±7.05 | 89.3±7.91 | 29.30±7.91 | 1.03±0.33 | 0.75±0.24 | 0.60±0.05 |
| A0A6A4LCV7 | 182.47±5.52 | 87.87±5.64 | 29.67±5.64 | 1.00±0.09 | 0.59±0.10 | 1.89±0.34 |
| A0A6A4LC04 | 76.8±2.42 | 201.97±2.93 | 21.23±2.93 | 1.00±0.02 | 7.42±0.40 | 2.40±0.17 |
| A0A6A4MA31 | 166.4±4.07 | 62.93±6.05 | 70.7±6.05 | 1.04±0.38 | 0.22±0.05 | 0.10±0.01 |
| A0A6A4LU02 | 162.53±1.17 | 72±1.73 | 65.43±1.73 | 1.02±0.28 | 1.23±0.15 | 3.93±0.46 |
| A0A385Z7Z4 | 141.63±2.35 | 100.23±1.17 | 58.17±1.17 | 1.00±0.01 | 0.45±0.03 | 1.03±0.11 |
| J7MFJ1 | 141.7±0.72 | 97.83±1.80 | 60.47±1.80 | 1.00±0.04 | 0.70±0.02 | 0.23±0.01 |
