# Supplementary figures and images for: Comparative Analysis of the Morphological, Physiological, Proteomic, and Metabolic Mechanisms of the “Biloxi” Blueberry Response to Shade Stress
Source: Front Plant Sci. 2022 May 3;13:877789. doi: 10.3389/fpls.2022.877789 (PMC9111170; doi:10.3389/fpls.2022.877789)

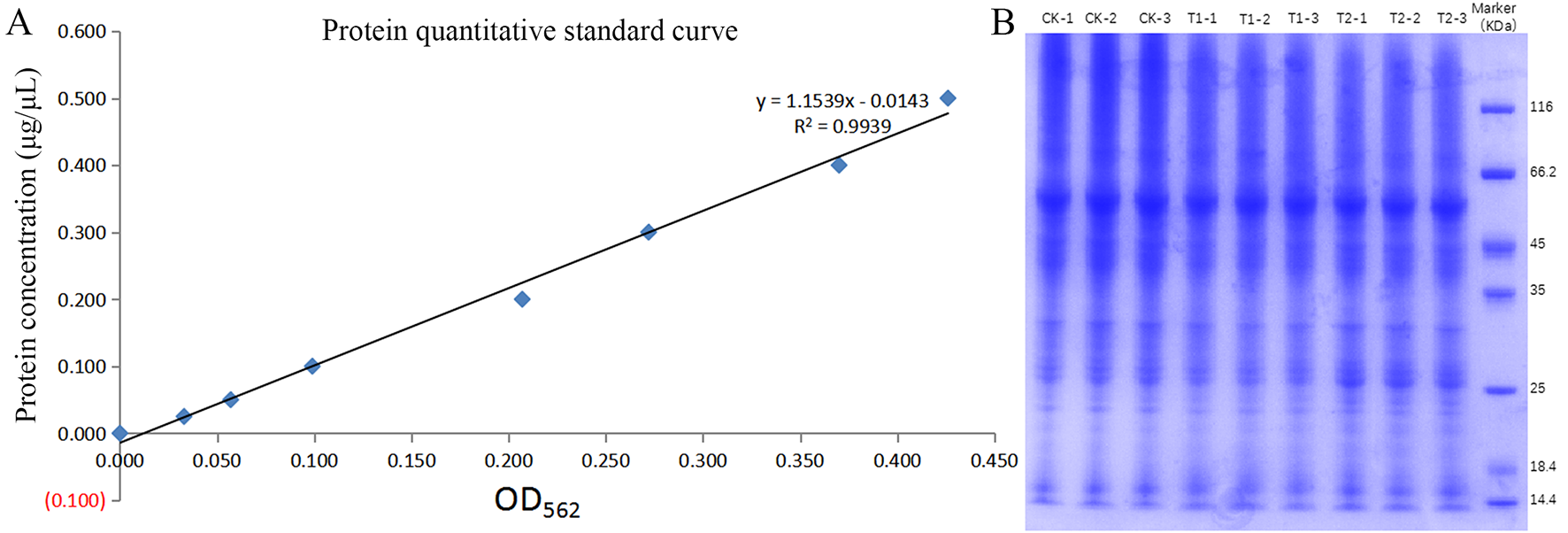

Supplement: Supplementary Figure 1 — Protein quantitative standard curve (A) and SDS–PAGE analysis of samples (B). [file Image_1.TIF]

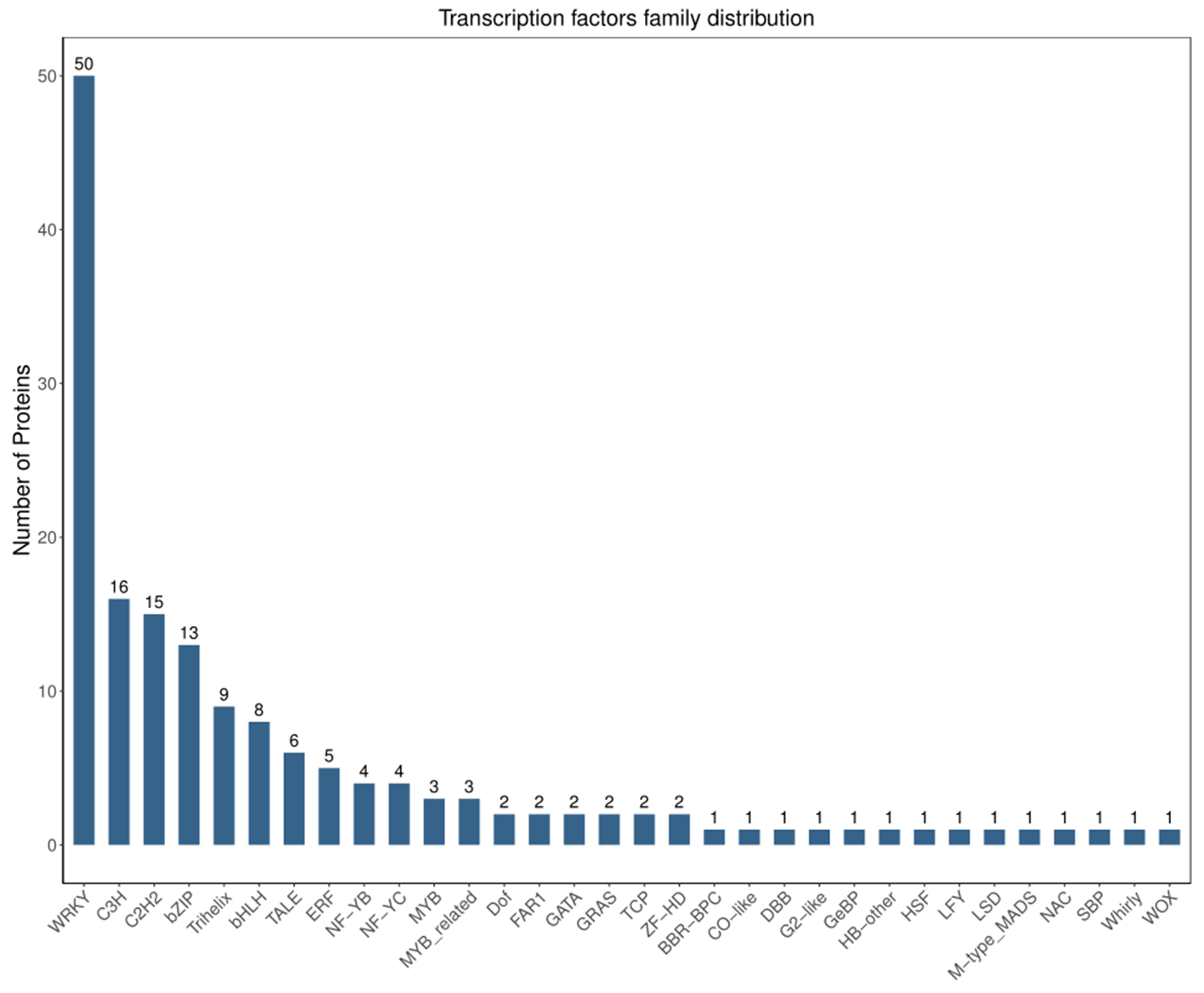

Supplement: Supplementary Figure 2 — Distribution of the transcription factor family in blueberry leaves. [file Image_2.PNG]

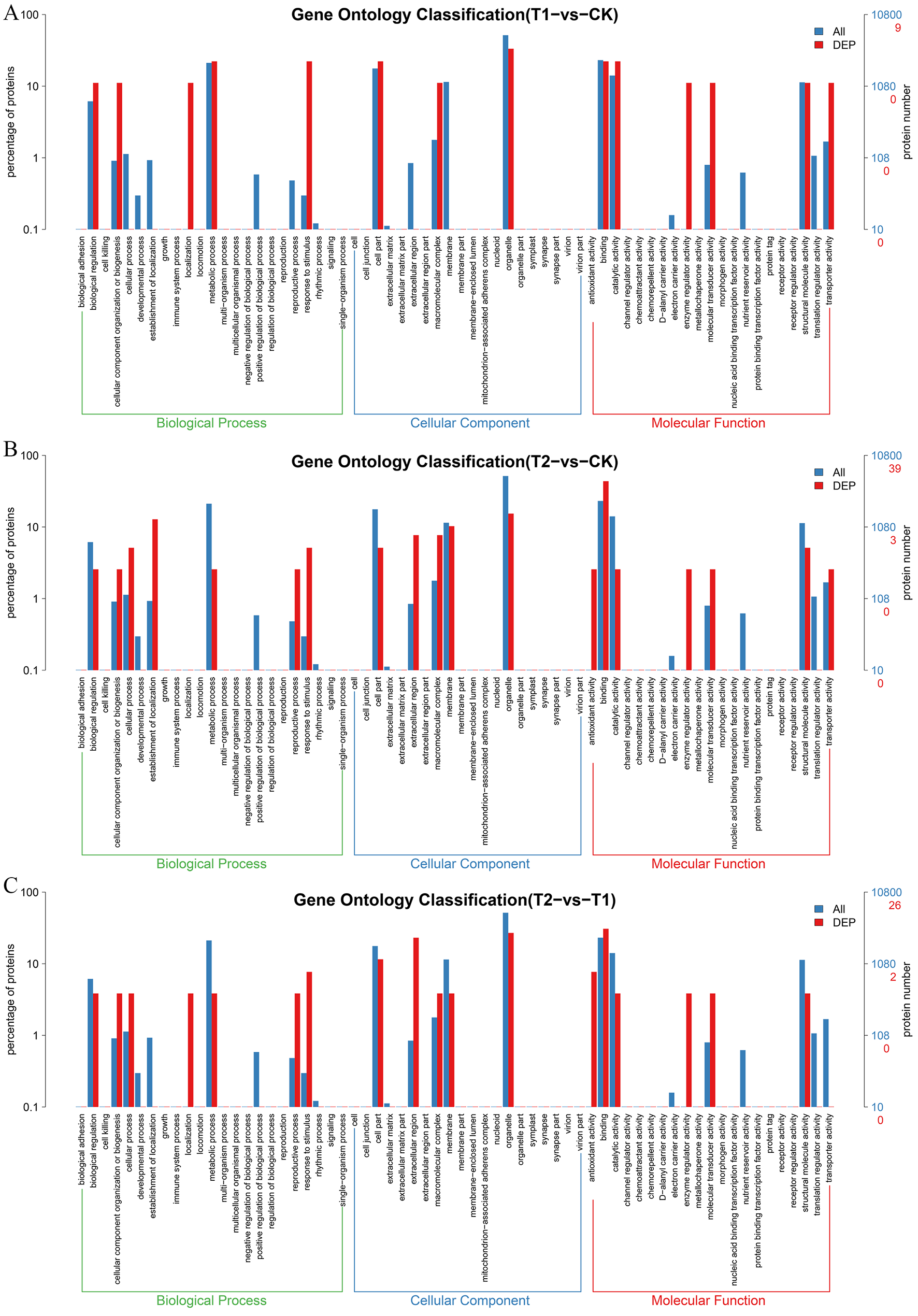

Supplement: Supplementary Figure 3 — Comparison of the differentially expressed proteins and all blueberry proteins at Gene Ontology classification level 2. [file Image_3.TIF]
